# Coenzyme M: An Archaeal Antioxidant as an Agricultural Biostimulant

**DOI:** 10.3390/antiox14020140

**Published:** 2025-01-24

**Authors:** Jeremy H. Brown, Jithesh Vijayan, Aline Rodrigues de Queiroz, Natalia Figueroa Ramos, Nate Bickford, Melissa Wuellner, Nicole R. Buan, Julie M. Stone, Katarzyna Glowacka, Rebecca L. Roston

**Affiliations:** 1 Department of Biochemistry, University of Nebraska-Lincoln, Lincoln, NE 68588, USA; jbrown353@huskers.unl.edu (J.H.B.); nbuan@unl.edu (N.R.B.); juliemstone2@gmail.com (J.M.S.); kglowacka2@unl.edu (K.G.); 2 Nebraska Center for Plant Science Innovation, University of Nebraska-Lincoln, Lincoln, NE 68588, USA; 3 Department of Natural Sciences, Oregon Institute of Technology, Klamath Falls, OR 97601, USA; nate.bickford@oit.edu; 4 Department of Biology, University of Nebraska at Kearny, Kearney, NE 68849, USA; wuellnermr@unk.edu; 5 Nebraska Center for Redox Biology, University of Nebraska-Lincoln, Lincoln, NE 68588, USA; 6 Institute of Plant Genetics, Polish Academy of Sciences, 60-479 Poznań, Poland

**Keywords:** Coenzyme M, antioxidant, photosynthesis, Arabidopsis, tobacco, basil, cannabis, soybean

## Abstract

Rising global food demand necessitates improved crop yields. Biostimulants offer a potential solution to meet these demands. Among them, antioxidants have shown potential to improve yield, nutritional quality, and resilience to climate change. However, large-scale production of many antioxidants is challenging. Here, we investigate Coenzyme M (CoM), a small, achiral antioxidant from archaea, as a potential biostimulant, investigating its effects on growth and physiology. CoM significantly increased shoot mass and root length of the model plant, *Arabidopsis thaliana*, in a concentration-dependent manner. Sulfur-containing CoM supplementation restored growth under sulfur-limited conditions in Arabidopsis, whereas similar recovery was not observed for other macronutrient deficiencies, consistent with it being metabolized. In tobacco, CoM increased photosynthetic light capture capacity, consistent with observed growth improvements. Interestingly, this effect was independent of carbon capture rates. Furthermore, CoM promoted early-stage shoot growth in various crops species, including tobacco, basil, cannabis, and soybean. Our results suggest CoM is a promising, scalable biostimulant with potential to modify photosynthesis and enhance crop productivity.

## 1. Introduction

Current models of population growth project a 35 to 56% increase in global food demand by 2050, necessitating improved crop yield efficiency to meet food security goals [1]. Application of biostimulants directly to field crops or even specialty crops produced under controlled environments (greenhouse or hydroponics) may provide an ideal solution [2]. One mode of action of biostimulants is through the modulation of reactive oxygen species (ROS) [3]. ROS is a key component of signaling, particularly for responses to abiotic and biotic stresses, and for plants specifically, ROS signaling interacts with photosynthesis and germination, among multiple other roles [4]. As such, antioxidants can act as biostimulants, and their capacity to confer conditional growth benefits have been reported across a wide variety of plant species under an array of environmental stress conditions [5,6,7]. Mechanisms for how antioxidants increase plant growth remain unclear; however, modification of redox homeostasis and amelioration of oxidative stress are hypothesized to be primary determinants in facilitating the growth enhancements attributed to exogenous antioxidants [6,7]. Additional rationales for improved growth suggest that applied antioxidants increase photosynthetic output by interacting with redox-responsive photosynthetic components. Indeed, modulation of photosynthetic parameters has been observed in response to exogenous application of antioxidants under control and stress conditions [7,8].

Photosynthesis has long been a target for improved crop efficiency, as improvements to photosynthetic capacity are often linked to improved productivity and yield [9,10,11,12]. Photosystem II (PSII) is often the target for photosynthesis improvements, since it is responsible for primary light capture. The maximum quantum yield (*F_v_*/*F_m_*) and efficiency of PSII (ϕ*PSII*) are parameters that are affected strongly by the buildup of ROS, which can occur readily under abiotic stresses such as high light, where PSII is unable to accommodate for excess electrons from light capture [13]. Nonphotochemical quenching (NPQ) of chlorophyll fluorescence is one way that plants have to disperse excess energy in the form of heat prior to the buildup of ROS, although the specific mechanisms for NPQ are not fully elucidated [14,15]. Excess energy not dissipated by NPQ or other means results in the generation of ROS within the chloroplast, which must be quenched or will result in the disassembly of light harvesting complexes through oxidative damage [16]. This ROS can be quenched through antioxidant pathways like the ascorbate–glutathione cycle [17]. The result of PSII energy use is the production of a proton gradient, ATP, and reducing equivalents, which are used in the Calvin–Bensen–Bassam cycle to fix carbon dioxide and transform captured light energy into chemical energy [18]. Application of antioxidants has caused variable effects on photosynthetic fluorescent parameters, but there is less information on how antioxidant application affects gas exchange and carbon fixation [19,20].

**Figure 1:**
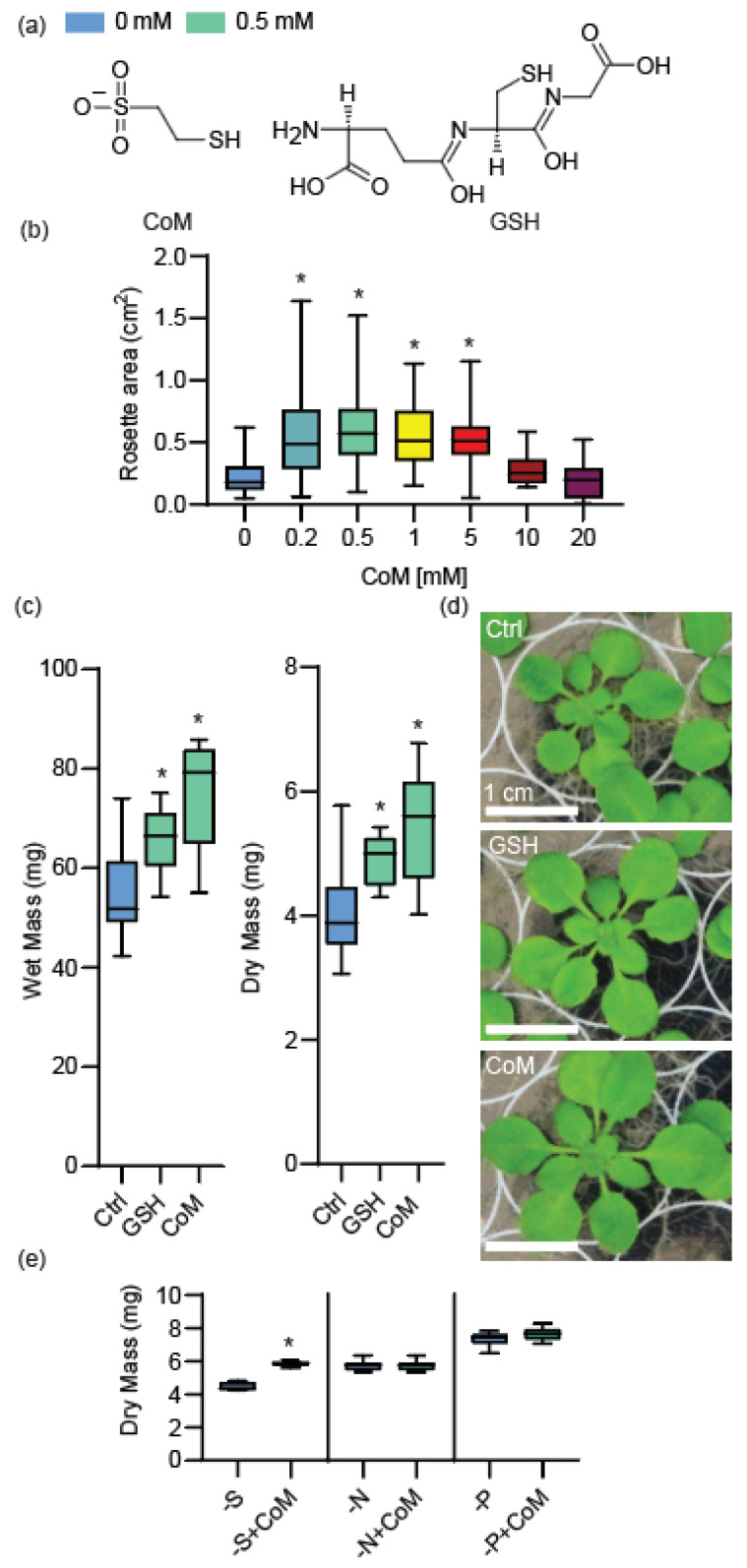
Growth and nutrient limitation effects of Coenzyme M application in Arabidopsis. (**a**) Structure of Coenzyme M (CoM) and Glutathione (GSH). (**b**) Rosette area measurements of Arabidopsis (at 25 days) grown on full-strength MS supplemented with CoM, *n* ≥ 41 biological replicates over ≥ 2 trials. (**c**) Dry mass measurements of Arabidopsis grown for 21 days on solid full-strength media supplemented with 0.5 mM of CoM or GSH. Mass on a per-plant basis with four measurements per trial for three trials, *n* ≥ 8 biological replicates. (**d**) Photographs of Arabidopsis from (**c**), scale bar is 1 cm. (**e**) Dry mass of Arabidopsis grown for 21 days with or without 0.5 mM of CoM on half-strength MS media formulated without sulfur, nitrogen, or phosphorus as indicated. *n* ≥ 7 biological replicates. Asterisks indicate *p*-value ≤ 0.05.

Coenzyme M (CoM) is a small antioxidant isolated from archaea, where it serves as a key component for methane production via the Wolfe cycle [21]. CoM’s structure is comprised of a sulfonate group connected to a thiol by a two-carbon bridge (Figure 1a) and is widely used as a human cancer medication under the drug name Mesna [22]. Mesna/CoM serves as a chemoprotectant against acrolein, a urotoxic metabolite that can be excreted into the urinary tract in response to some chemotherapy treatments. Mesna/CoM’s potent chemoprotective property stems from the reactivity of CoM’s thiol (-SH) group with alkylating groups of acrolein, forming inactive thioethers in the process [22]. The reactivity of CoM’s thiol group also gives CoM its reductive capacity, allowing it to act as an antioxidant. Thiols are highly reactive with electrophiles and oxidants, acting as reductants by donation of their thiol hydrogen [23]. CoM is achiral, rendering it relatively easy to produce in abundance through chemical synthesis with 1,2-dichloroethane as the starting material [24]. Its structural simplicity and synthetic scalability position CoM as a candidate for agricultural applications, particularly when evaluated against natural antioxidants like glutathione (GSH), which share similar properties but face production limitations.

CoM is of archaeal origin and not endogenously produced in humans nor plants, and no former work has been carried out on its interactions with plants. The closest comparable endogenous antioxidant to CoM in plants is Glutathione (GSH), which also contains a thiol group to confer redox activity (Figure 1a). CoM and GSH share similar reduction potentials of −271 and −264 mV, respectively [25,26]. GSH’s functions in plants have been studied extensively, and when applied exogenously or upregulated internally, it has biostimulant properties [27,28,29]. GSH’s many roles include acting as a signaling intermediate for stress responses, detoxifying metals and xenobiotic materials, and maintenance of ROS homeostasis through the ascorbate/GSH cycle, among other important roles [30]. When applied exogenously, it restores chlorophyll content [28], reduces levels of reactive oxygen species [28,31], and protects membranes [28,32], among other functions (reviewed in [32].

One of the primary differences between CoM and GSH is that GSH is both chiral and a tripeptide, making it difficult to produce in abundance [33]. A major hurdle in the widespread use of antioxidants in agriculture is that large-scale antioxidant(s) synthesis/production can be challenging due to the fact that effective antioxidants often possess multiple chiral centers, hindering mass production [33,34,35]. If CoM has similar positive effects on growth of various plant species as has been reported for GSH [7], it could be an economical, readily synthesized, exogenously applied effective alternative to increase plant growth, resilience, and crop yield.

In this study, we investigate the effects of CoM application on several plant species to determine its potential as an agricultural biostimulant. CoM was applied to the model species, *Arabidopsis thaliana*, as well as to multiple agriculturally relevant species. Improved plant growth, including aerial and root tissue biomass and plant height, were observed in response to exogenous CoM application. Moreover, CoM treatment elicited significant changes in a range of photosynthetic parameters that may contribute to our observed growth enhancements.

## 2. Materials and Methods

### 2.1. Plant Growth

Arabidopsis: *Arabidopsis thaliana* (Columbia ecotype) was grown on solid full-strength or half-strength Murashige and Skoog (MS) medium with vitamins (Caisson Labs, Smithfield, UT, USA) and media (pH 5.7) supplemented with CoM (AstaTech, Bristol, PA, USA) or GSH (Acros Organics, Waltham, MA, USA) at various concentrations. For nutrient limitation experiments, formulations of MS without N, P, or S (Caisson Labs) were used. Seeds were bleach-sterilized, rinsed, and sown on media plates. Plates were placed in 4 °C for 48 h to break seed dormancy and then transferred to a Percival CU36L4 growth chamber with a 16 h light/8 h dark photoperiod, with 22 °C day at 150 µmol m^−2^ s^−1^ light and 18 °C night.

Tobacco: *Nicotiana tabacum* was grown on soil under greenhouse conditions. Seeds were sown on a greenhouse mixed soil (8:8:3:1 (*w*/*w*/*w*/*w*) peat moss:vermiculite:sand:screened topsoil, with 7.5:1:1:1 (*w*/*w*/*w*/*w*) Waukesha fine lime, Micromax, Aquagro, and Green Guard per 0.764 m^2^). Plants were grown in a greenhouse with a 16 h light/8 h dark photoperiod with 23–25 °C day and 18–20 °C night.

Basil: *Ocimum basilcum* v. Genovese (basil) seeds were germinated on wet paper towels until radicles emerged, all seeds were transplanted to greenhouse mixed soil within two days. Plants were grown in a greenhouse with a 16 h light/8 h dark photoperiod with 20–23 °C day and 18–20 °C night.

Cannabis: *Cannabis sativa* plants were clonally propagated from a single individual for the study and grown in greenhouse mixed soil. Greenhouse supplemental lighting was provided to generate a 16 h light/8 h dark photoperiod with temperatures set between 20 and 23 °C in the day and between 18 and 20 °C in the night.

Soybean: *Glycine max* (Thorne) seeds were sown on a greenhouse mixed soil. Plants were grown in a greenhouse with a 16 h light/8 h dark photoperiod with 23–25 °C day and 18–20 °C night. Plants were grown to 21 days after sowing, with biweekly spray treatment beginning at 7 days post-sowing.

All plants grown on soil were fertigated once per week by application of NPK 20-10-20 (JR Peters, Inc., Allentown, PA, USA) diluted to 200 parts per million through sub-irrigation of the pot.

### 2.2. CoM Application

Solid media: In experiments where plants were grown on solid Murashige and Skoog (MS) media (as described by [36], filter-sterilized (0.2 µm syringe filters, Whatman, Florham Park, NJ, USA) CoM or GSH dissolved in water at concentrations indicated in figure legends were added to autoclaved MS media solutions at approximately 50 °C immediately prior to solidification. An amount of 100 mL of media was solidified in 150 × 25 mm Petri dishes (Sarstedt, Newton, MA, USA). CoM and GSH used for these experiments were prepared by fresh dilution and sterilization from powdered forms stored under N_2_ at 4 °C.

Spray: In experiments where CoM was applied via spray application, fresh solutions of CoM in MilliQ water at concentrations indicated in figure legends were prepared immediately prior to application. Mixing of CoM was performed with minimal agitation, and it was sprayed by a hand sprayer, forming droplets no larger in diameter than 2 mm. CoM solutions were sprayed to cover the majority of the adaxial surfaces of the leaves and allowed to dry in the plant’s growth chambers.

Seed soak: In experiments where CoM was applied via seed soaking, solutions of CoM in MilliQ water at concentrations indicated in figure legends were prepared and seeds were soaked at room temperature overnight (14 h) prior to planting. The volume of solution was adjusted to cover the seeds.

### 2.3. Plant Tissue and Size Measurements

Tissue collection: Aerial shoot and leaf tissues were collected and weighed to measure fresh mass; then, tissue was dried at 80 °C for 3–4 days in an Avantco CO-16 convection oven to measure dry mass. Mass measurements were carried out on an Ohaus Pioneer Analytical Balance PA-64. For Arabidopsis experiments on solid media, multiple individual plants were aggregated, and data are presented on a per-plant basis. Measurements for tobacco, soybean, and other species are represented on the basis of individual plants.

Rosette size measurements: Top-down photos of Arabidopsis were taken with a 1 cm^2^ size marker. Rosette size was calculated using the easy leaf area method [37]. Overlapping plants were removed at 25 days of growth.

Height and area measurements: Height from the base of the stem to the highest point of the aerial tissues was recorded. To determine approximate area, plant height was multiplied by the width of aerial tissues at their widest point.

### 2.4. Sulfur Analysis

Arabidopsis shoot tissue was collected after 21 days of growth, and 50 mg of aerial tissue was used for ICPMS analysis. Intracellular sulfur content was measured by inductively coupled plasma mass spectrometry (ICPMS) as described in [38,39].

### 2.5. Photosynthetic Analyses

To measure chlorophyll fluorescence, plants were submitted to a pulse-amplitude-modulated protocol during 10 min of light illumination at 800 μmol m^−2^ s^−1^, followed by 10 min of darkness, using a chlorophyll fluorescent imager (FluorCam FC 800-C). Saturating flashes of 2400 μmol m^−2^ s^−1^ for a duration of 800 ms were used in the light and dark periods. First, *F_o_* was measured in dark-adapted plants, followed by capture changes in steady-state fluorescence (*F_s_*) and maximum fluorescence under illuminated conditions (*F_m_*′) over time; the measurements were obtained at the following time points: 0, 0.25, 0.5, 0.75, 1, 2, 3, 4, 5, 6, 7, 8, 9, 10, 10.25, 10.5, 11, 12, and 15 min. The raw data were processed automatically with fluorescence background exclusion. Maximum quantum yield is defined as *F_v_*/*F_m_* in dark-adapted plants, and NPQ is defined as *F_m_*/(*F_m_*′ − 1). The data were fitted to hyperbola equations according to [40], where the induction rate Ind_(Rate)_ was the initial slope of the hyperbola in light, and the relaxation rate Rel_(Rate)_ was the initial slope hyperbola in the dark. For our dose–response curves, we performed a baseline correction to determine the percent change in those parameters relative to control measurements. Those results were fitted into a polynomial equation.

Gas exchange measurements were collected according to [40], using a LICOR 6800 (LICOR, Lincoln, NE, USA), with the exceptions that block temperature was set to 25 °C, [CO_2_] was set to 400 µmol, water vapor pressure deficit was set to 1.3 kPa, and maximum light intensity was set to 2000 µmol m^−2^ s^−1^. Curve fitting of rubisco carboxylation rate (V_cmax_), rate of electron transport (J), and triose phosphate use (TPU) were calculated according to [41].

### 2.6. Statistical Analyses

All statistical analyses were performed using Graphpad Prism v10.3.1. As indicated in Table S1, ANOVAs and corrected *t*-tests were performed as appropriate. To compare pair-wise values, one-way ANOVAs were followed by Dunnett’s multiple comparisons tests, or two-way ANOVAs were followed by Bonferroni’s multiple comparison test. Finally, for experiments with only two comparison groups, Welch’s corrected *t*-test, 2-tailed tests, and unpaired *t*-tests were used. Throughout the paper, *p*-values represent the adjusted *p*-value determined by the indicated statistical test with the number of biological replicates represented (*n*).

## 3. Results

### 3.1. Coenzyme M Improves Growth and Is Metabolized by Arabidopsis

As an initial investigation into CoM’s potential applicability to improve plant biomass, we used the model plant *Arabidopsis thaliana* ecotype Columbia (Arabidopsis). To determine optimal concentrations of CoM for application, seeds were grown on plates of solid synthetic growth media supplemented with CoM concentrations from 0.2 to 20 mM (Figure 1b). These concentrations were chosen after preliminary empirical tests implied that they would encapsulate the response curve for Arabidopsis. Results showed that concentrations of CoM between 0.2 and 5 mM significantly increased the rosette size of Arabidopsis at 25 days of growth (Figure 1b). The maximal enhancement reached was 168% (0.226 cm^2^ to 0.605 cm^2^) of untreated control plants with 0.5 mM of CoM at 25 days (*p*-values in Table S1). The presence of CoM did not appear to affect germination (Figure S1).

Similar to CoM, another thiol-based reductant, glutathione (GSH), has been applied to plants at comparable concentrations (0.05 to 1 mM) to investigate its impact on growth [27,28,29]. The effective range of applied concentrations shared between glutathione and CoM were from 0.2 to 1 mM of each antioxidant. To directly compare the effects of CoM and GSH, we assessed Arabidopsis biomass near the center of the CoM effect range after growth in two common synthetic media (full-strength and half-strength MS supplemented with 0.5 mM of either reductant). Both reductant treatments caused increased mass of aerial tissues at 21 days of growth relative to mock treatment (Figure 1c,d). The dry mass increased by 35% (4.025 mg to 5.425 mg) and 22% (4.025 mg to 4.897 mg) with CoM and GSH supplementation, respectively, in full MS medium. Fresh mass was increased similarly. Under half-strength MS conditions, the increase in Arabidopsis aerial tissue was only significant in fresh mass—30% (4.620 mg to 5.063 mg) for CoM and 17% (4.620 mg to 4.700 mg) for GSH—suggesting a media-dependent effect. To assess the root growth response to CoM, Arabidopsis were grown on vertical plates supplemented with 0.5 mM of CoM or GSH. CoM increased root length significantly versus control (by 47% (3.573 cm to 5.246 cm)), and while GSH treatment showed the same trend, with a 9.7% increase in average length (3.573 cm to 3.920 cm), it was not significant (Figure S2).

GSH is a naturally occurring antioxidant in plants, while CoM is not, but they both had similar impacts on growth in our assays. We wanted to understand the likelihood that CoM, like GSH, is taken up and metabolized. Because CoM contains sulfur, we investigated the possibility that it could provide this nutrient by testing the growth of plants in CoM supplementation in MS media reformulated to lack each of the major macronutrients: nitrogen, phosphorus, and sulfur. Arabidopsis grown on nitrogen- and phosphorus-limited media showed no significant changes with CoM supplementation, while those grown on sulfur-limited medium showed an increase in dry mass of 30.2% (4.471 mg to 5.859 mg) when supplemented with 0.5 mM of CoM (Figure 1e), suggesting that CoM was taken up and metabolized for sulfur. However, direct measurement of sulfur content in plants supplemented with CoM or GSH revealed no detectable differences compared to untreated controls (Figure S3), suggesting that supplementation supports growth under sulfur limitation without measurable increases in sulfur at this developmental stage.

### 3.2. Coenzyme M Increases Photosynthetic Capacity

**Figure 2:**
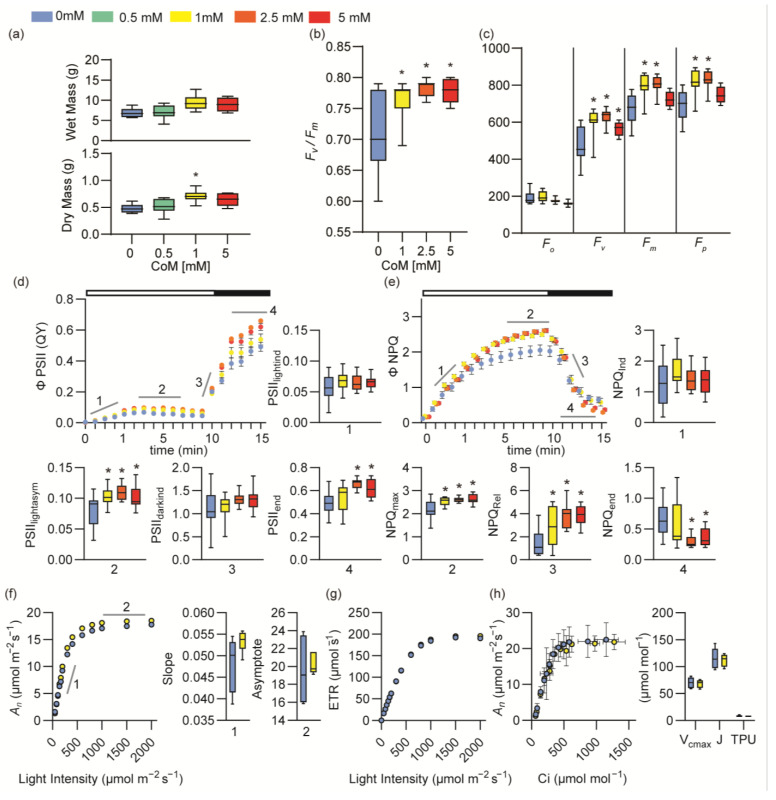
Photosynthetic parameter effects of Coenzyme M application in tobacco. (**a**) Fresh and dry mass measurements of tobacco grown in soil for 21 days with aerial tissues sprayed with CoM twice weekly, *n* = 6 biological replicates. Identically grown tobacco plants were tested for their photosynthetic traits as measured by FluorCam FC 800-C in panels (**b**–**h**). (**b**) The maximum quantum yield, *F_v_*/*F_m_*, measured after dark adaptation, *n* = 12 biological replicates. (**c**) Measurements of minimum (*F_0_*), maximum (*F_m_*), or variable fluorescence (*F_v_*, *F_p_*) after dark adaptation, *n* = 12 biological replicates. (**d**) Photosystem II operating efficiency (ϕPSII) measured after biweekly spray of water; 1 mM, 2.5 mM, or 5 mM of CoM; and dark adaptation. Component traits of ϕPSII are indicated by gray lines and numbers and represented in separate graphs: (1) PSII_lightind_, the initial slope of a linear fit including the first two points and zero in the light period (indicated by the white bar at the top of the ϕPSII graph); (2) PSII_lightasym_, the hyperbolic asymptote during the light period; (3) PSII_darkind_, the slope of the first two points of PSII induction in the dark period (indicated by the black bar at the top of the ϕPSII graph); and (4) PSII_end_, the hyperbolic asymptote during the dark period, *n* = 12 biological replicates. (**e**) Nonphotochemical quenching (ϕNPQ) measured under the same conditions as ϕPSII, with its component traits indicated by gray lines and numbers and represented in separate graphs. (1) NPQ_ind_, the initial slope of a linear fit including the first two points and zero in the light period; (2) NPQ_max_, the hyperbolic asymptote during the light period; (3) NPQ_rel_, the slope of the last light point and first two points of NPQ relaxation in the dark period; and (4) NPQ_end_, the hyperbolic asymptote during the dark period, *n* = 12 biological replicates. Panels (**f**,**g**) represent LiCOR 6800 measurements of 21-day-old tobacco plants sprayed biweekly with 1mM of CoM. (**f**) Carbon assimilation (*A_n_*) in response to increasing light, and its component parts, the initial slope (1) and hyperbolic asymptote (2), *n* = 6 biological replicates. (**g**) The electron transport rate (ETR), *n* = 6 biological replicates. (**h**) The internal concentration of CO_2_ (*C_i_*) in response to increasing light, and maximum carboxylation rate allowed by Rubisco (*V_cmax_*), the rate of photosynthetic electron transport based on NADPH requirement (*J*), and triose phosphate use (*TPU*) fit from the *C_i_* curve. All asterisks indicate *p*-value ≤ 0.05.

Increased plant growth is often linked to changes in photosynthetic parameters [9,10,12,15]; therefore, to investigate whether CoM treatment influenced photosynthesis, we conducted greenhouse trials on *Nicotiana tabacum* (tobacco), a common model for photosynthesis studies [42,43]. We began by verifying the effect of CoM on growth. Tobacco sprayed with CoM at concentrations between 0.5 and 5 mM significantly increased biomass in a concentration-dependent manner (*p* = 0.0114, one-way ANOVA). (Figure 2a and Figure S4). The maximum increase in arial tissue dry mass was 48% (0.874 g to 1.006 g) with 1 mM spray application, with a second trial confirming the trend of 1 mM resulting in the most significant increase in plant biomass (Figure S4). To determine whether CoM treatment influenced photosynthesis, we examined multiple parameters in dark-adapted tobacco sprayed with CoM at concentrations of 1, 2.5, and 5 mM. The maximum efficiency of photosystem II (*F_v_*/*F_m_*) increased with CoM treatment by 7.6% (0.710 to 0.783) with 1 mM of CoM (*p* = 0.0001, one-way ANOVA; Figure 2b). *F_v_*/*F_m_* is calculated from multiple components; thus, we checked if minimum (*F*_0_), maximum (*F_m_*), or variable fluorescence (*F_v_*, *F_p_*) was the largest contributing component. We found that *F_m_*, *F_v_*, and *F_p_* each increased after CoM treatment, consistent with an increase in the number of open photosystem II reaction centers (Figure 2c). We then challenged dark-adapted plants with an actinic light and pulses to saturate photosynthetic capacity and activate regulated energy dissipation measured as non-photochemical quenching (NPQ) followed by dark relaxation. Under these conditions, application of CoM increased the operating quantum yield of photosystem II (*ϕPSII*) at the end of both the light (Figure 2d2) and dark (Figure 2d4) periods, though it did not affect the rates of induction (Figure 2d1) or relaxation (Figure 2d3). Similarly, CoM increased the maximum NPQ in the light period (Figure 2e2) and the rate of NPQ relaxation (Figure 2e3) and residual NPQ in the dark period (Figure 2e4). The observed increases in open photosystem II reaction centers (*F_m_*, *F_v_*, and *F_p_*) and yield of photosystem II (*ϕPSII*) and the increased rate of NPQ relaxation are all consistent with increased energy capture.

To explore the effect of CoM on carbon capture, we measured gas exchange parameters in response to increasing light and CO_2_ concentrations using an open gas exchange system (LICOR 6800). We found that tobacco treated with CoM seemed to have a slight increase relative to controls in net carbon assimilation (*A_n_*) during both light-limited (slope) and light-saturated (asymptote) portions of the curve, though the effect was too small to be statistically significant at the 95% confidence interval (Figure 2f). We also checked for CoM treatment impacts on the electron transport rate or CO_2_ usage and found that neither were affected (Figure 2h). Specifically, we measured how carbon assimilation varied with the change in CO_2_ dosage, and we estimated limiting factors by curve fitting, defining the effect on the maximum carboxylation rate allowed by Rubisco (*V_cmax_*), on the rate of photosynthetic electron transport (*J*), and on the triose phosphate use (*TPU*), none of which were impacted by CoM treatment. Stomatal conductance (*G_s_*) was not significantly affected by CoM application (Figure S5).

### 3.3. CoM Improves Growth of a Range of Crop Species

**Figure 3:**
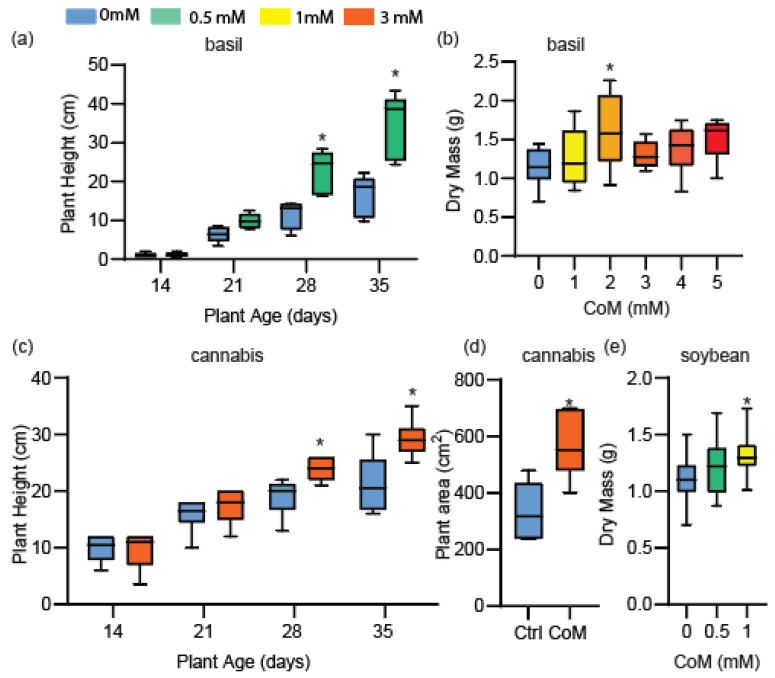
Growth effects of Coenzyme M application in various plant species—basil, cannabis, and soybean. (**a**) Plant height measurements of basil at ages indicated below. Seeds were soaked in 0.5 mM of CoM prior to sowing, and plants were sprayed once weekly with 0.5 mM of CoM, *n* = 5 biological replicates. (**b**) Dry mass measurements of basil after 30 days of growth during which CoM at concentrations indicated below was sprayed three times a week, *n* = 9 biological replicates. (**c**) Plant height measurements of cannabis at indicated ages that were sprayed three times weekly with 3 mM of CoM, *n* = 6 biological replicates. (**d**) Plant area measurements of cannabis sprayed three times weekly with 3 mM of CoM, *n* = 6 biological replicates. (**e**) Dry mass measurements of soybean seedlings after 21 days of growth on soil during which aerial tissues were sprayed with CoM twice weekly, *n* = 30 biological replicates. All asterisks indicate *p*-value ≤ 0.05 All mass measurements represent aerial tissues.

Model plants Arabidopsis and tobacco both showed improved growth as a result of CoM application, therefore we applied CoM to more agriculturally relevant species to explore potential use in an agricultural capacity. In two trials we used *Ocimum basilcum* v. Genovese (basil, Figure 3a,b). Basil showed a height increase after 28 (98%, 11.40 cm to 22.56 cm) and 35 days (111%, 16.28 cm to 34.34 cm) of growth when seeds were soaked with 0.5 mM of CoM and sprayed once weekly with 0.5 mM of CoM (Figure 3a). To test the concentration-dependence of CoM, CoM was applied to basil by foliar spray twice weekly with concentrations between 1 and 5 mM. The greatest increase in basil dry mass was observed after treatment with 2 mM of CoM (39.9%, 1.143 g to 1.599 g; Figure 3b). *Cannabis sativa* (cannabis) received a foliar spray with 3 mM of CoM three times weekly. Height was increased at 28 days (25%, 19.00 cm to 23.86 cm) and 35 days (38%, 21.33 cm to 29.43 cm) of growth relative to control plants (Figure 3c). The shoot area of these plants was also increased at 35 days of growth (69%, 335.67 cm^2^ to 565.71 cm^2^; Figure 3d). To broaden the potential applicability of CoM treatment to commodity crops, we also tested *Glycine max* v. Thorne (soybean). After leaflets emerged, they received a foliar spray of 0.5 or 1 mM of CoM twice a week. Aerial tissue dry mass was increased (17.3%, 1.119 g to 1.313 g) after 21 days of growth (stage V2–V3) with 1 mM of CoM application (Figure 3e). We concluded that CoM application stimulated growth of multiple species following frequent foliar spray.

## 4. Discussion

Our results showed that the application of CoM enhanced growth in a species-specific and concentration-dependent manner. Arabidopsis, tobacco, basil, cannabis, and soybean all showed increased growth parameters with at least one concentration of CoM tested. Additionally, multiple growth parameters were affected by CoM application, including wet and dry mass of aerial tissues (Figure 1c, Figure 2a and Figure 3b,e), shoot height (Figure 3a,c), leaf area (Figure 1b and Figure 3d), and root growth (Figure S2). Many of these parameters were increased similarly in diverse plant species. In tobacco, we determined that CoM was directly influencing light energy capture, a likely cause of improved growth (Figure 2b–e). Using CoM to increase the growth of aerial leaf tissues would be particularly beneficial for crop species like tobacco and basil, since their yield is leaf tissue. It is also likely to benefit soybean and other seed crops in which final yield is related to initial growth [44].

### 4.1. Mode of CoM Action

Antioxidants may have different roles on the surface of plants and after internalization [7]. Since the ability of plants to internalize CoM was unknown, we investigated its potential to provide a source of sulfur, finding that CoM improved biomass of Arabidopsis grown in media lacking S, but not media lacking N or P (Figure 1e). This is consistent with CoM being internalized and metabolized as a source of sulfur, though we cannot rule out the possibility that this effect is indirect. We attempted to measure the internal concentration of sulfur; however, neither GSH nor CoM treatment measurably increased the sulfur content of Arabidopsis in our growth conditions (Figure S3). GSH is generally considered to be internalized by Arabidopsis due to the increase in internal GSH after application [29] and the phenotypic similarity of external application and internal upregulation [45]. Since GSH application did not result in measurable S increases, and previous studies have found that GSH application does not always increase measurable sulfur depending on species, we conclude that CoM’s role as a metabolizable sulfur source remains plausible [46].

Since photosynthesis is strongly affected by redox status and previous studies have found that antioxidant application can influence photosynthetic parameters, we investigated how CoM affects photosynthesis as a mode of action for increased growth [19,20]. Application of CoM increased the maximum yield of photosystem II in both light and dark conditions (Figure 2b–d) but, interestingly, did not influence carbon assimilation in a significant way (Figure 2f). These effects are consistent with the increase in tobacco growth (Figure 2a). Additionally, NPQ_max_ and NPQ relaxation rate were improved with CoM application (Figure 2e), suggesting a greater capacity to dissipate excess energy from light capture and return to a more efficient energy utilization state faster. Effects on NPQ rate were previously linked to growth increases [9]. It is also an interesting result in the context of antioxidant application, as previous studies have shown that antioxidants have variable effects on NPQ [7,47,48]. CoM application on tobacco simultaneously increased the plant’s ability to perform photosynthesis by increasing the amount of active PSII centers while also increasing its capacity to dissipate excess energy associated with high light in the form of NPQ, an uncommonly reported dual benefit for antioxidant application. Unexpectedly, these changes are independent of rubisco carboxylation (*V_cmax_*) and electron transport rate (*J*) (Figure 2g,h), which other antioxidants have influenced [43,49]. We conclude that changes in photosynthesis caused by application of CoM are consistent with observed increases to growth parameters and that CoM affected photosynthesis uniquely compared to application of other antioxidants like GSH [19].

### 4.2. CoM Impacts on Growth

Benefits derived from the application of antioxidants are almost always concentration-dependent [7,50]. Applying a substance, antioxidant or otherwise, in sufficient concentration will have detrimental effects on growth and health of the plant treated [51]. For instance, melatonin applied at 100 mM inhibited root growth of *Brassica juncea*, but when it was applied at 0.1 mM, it promoted root growth [52]. We found that CoM in concentrations between 0.2 and 5 mM had a positive effect on the growth of Arabidopsis, while concentrations of 10 mM and above had neutral or detrimental effects (Figure 1d). Effective concentrations appear to be species-specific, as tobacco and soybean did not share an effective concentration of CoM (Figure 2a and Figure 3e). Interestingly, when looking at agriculturally relevant species, CoM had a narrower range of effective concentration (Figure 2a and Figure 3b,e) compared to Arabidopsis (Figure 1b). This could be a byproduct of the application method, as we applied CoM to Arabidopsis through media supplementation, while other species received a foliar spray.

### 4.3. Potential for CoM Use as a Biostimulant

Agriculturally, biostimulants have been applied to seeds, plants, and soil through seed soaking or coating, foliar spray, or soil dry or wet applications, with the most common of these being foliar spray [53]. We mainly explored CoM application by foliar spray to agriculturally relevant species (Figure 2 and Figure 3). We found that it could provide a growth increase in tobacco up to 48% (0.874 g to 1.006 g), in basil up to 39.9% (1.143 g to 1.599 g), and in soybean up to 17.3% (1.119 g to 1.313 g) (Figure 2a and Figure 3b,e). It is important to note that the effective concentration of a biostimulant will be influenced by which plant it is being applied to and the method by which it is being applied [7,54]. For CoM, we found that the most effective concentrations differed between tobacco and basil (Figure 2a and Figure 3b). Previous studies with biostimulants also showed that environmental conditions can have a drastic effect on the efficacy of biostimulant application [55,56]. Trial-to-trial variation in tobacco suggested that CoM affects an aspect of plant growth that fluctuated with its environment. For example, two growth trials separated by three months resulted in different increases in dry mass, with one at 48% (0.874 g to 1.006 g) (Figure 2a) and the other at 15.1% (0.479 g to 0.709) (Figure S4). These trials also had different dry masses of control plant growth, presumably due to seasonal differences in day length and temperature, which is known to impact growth in the presence of supplemental lighting and heating [57,58].

ROS and the maintenance of ROS homeostasis are key signaling components for many growth processes [59]. As such, altering ROS homeostasis through the addition of an antioxidant could introduce problems through the disruption of ROS signaling. An important consideration for the viability of an antioxidant as a biostimulant is whether it disrupts germination, since germination is heavily influenced by redox signaling [60]. We found that CoM did not detrimentally affect the germination of Arabidopsis sown on media containing 0.5 mM of CoM. However, the roles of ROS signaling are far-reaching and include biotic and abiotic stress responses, diurnal cycles, opening and closing of stomata, and many other key cellular functions [61]. An example of a key ROS function is the oxidative respiratory burst caused by signaling of a pathogenic invasion [59,62]. Our study did not exhaustively test the many potential ROS responses, and thus, the possibility of a negative impact due to CoM-induced redox imbalance remains.

Finally, ease of synthesis is an aspect that is important to the economic value and availability of a biostimulant [63]. GSH is a chiral molecule, and synthesis can be achieved through an enzymatic method in yeast which allows for the purification of GSH with the correct chirality [33]. Since CoM does not have chiral centers, it can be chemically synthesized without a biological host and, thus, at larger scales. One method of synthesizing CoM uses 1,2-dichloroethane as starting material, which can readily be synthesized from ethanol [24].

The ability to produce CoM in large quantities, a lack of disruption in germination and health of plants, and the ability to improve growth suggests that CoM has the potential to be an effective and low-cost biostimulant.

## Data Availability

Data is contained within the article or Supplementary Materials.

## Supplementary Materials

The following supporting information can be downloaded at: https://www.mdpi.com/article/10.3390/antiox14020140/s1, Figure S1. Germination rate of Arabidopsis is unaffected by CoM application. Germination rates of Arabidopsis grown on solid media supplemented with 0.5 mM GSH or CoM, *n* = 3 biological replicates. Lack of asterisks indicates no *p*-value ≤ 0.05. Figure S2. Root growth of Arabidopsis is positively affected by CoM application. Root growth of Arabidopsis grown on solid media supplemented with 0.5 mM GSH of CoM, *n* = 3 biological replicates. All asterisks indicate *p*-value ≤ 0.05. Figure S3. Intracellular sulfur content of Arabidopsis is unaffected by CoM application. Measurements of internal S_34_ content of Arabidopsis grown on solid media supplemented with 0.5 mM GSH or CoM or 0.3 mM of MgSO_4_ × 7H_2_O, *n* = 3 biological replicates. All asterisks indicate *p*-value ≤ 0.05. Figure S4. Growth of tobacco is increased by CoM application. Second growth trial of tobacco grown to 21 days with spray application of CoM twice weekly with concentrations in mM listed. All asterisks indicate *p*-value ≤ 0.05. Figure S5. Stomatal conductance of tobacco is unaffected by CoM application. Stomatal conductance (*G_s_*) of tobacco grown to 21 days with spray application of 1 mM of CoM twice weekly, as measured by LiCOR 6800, in response to increasing light. *n* = 6 biological replicates; lack of asterisks indicate no *p*-value ≤ 0.05. Table S1. Statistical analyses of data.
