# Supplementary material for: Coenzyme M: An Archaeal Antioxidant as an Agricultural Biostimulant
Source: Antioxidants (Basel). 2025 Jan 24;14(2):140. doi: 10.3390/antiox14020140 (PMC11851959; doi:10.3390/antiox14020140)
Supplement: Supplementary file 1 [file antioxidants-14-00140-s001.zip › antioxidants-3413012-supplementary.pdf]

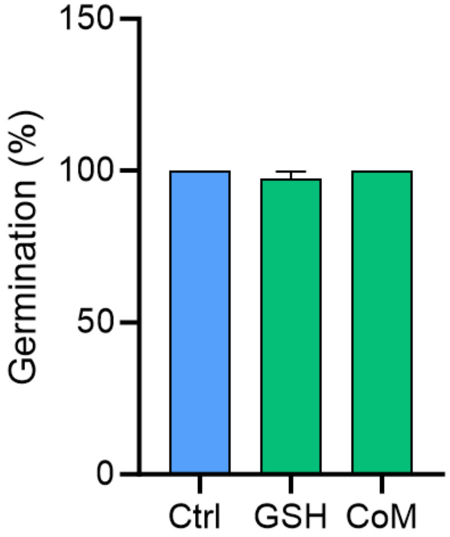

Figure S1. Germination rate of Arabidopsis is unaffected by CoM application. Germination rates of Arabidopsis grown on solid media supplemented with 0.5 mM GSH or CoM n = 3 biological replicates. Lack of asterisks indicates no p-value ≤ 0.05.

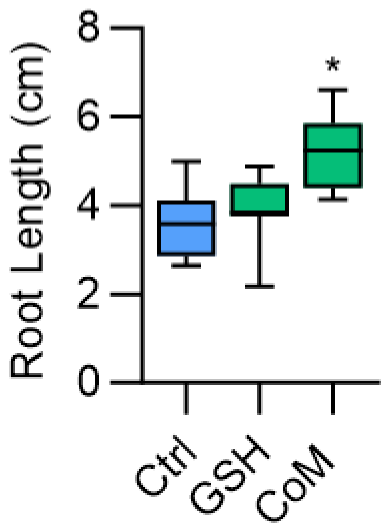

Figure S2. Root growth of Arabidopsis is positively affected by CoM application. Root growth of Arabidopsis grown on solid media supplemented with 0.5 mM GSH of CoM *n* = 3 biological replicates. All asterisks indicate p-value ≤ 0.05.

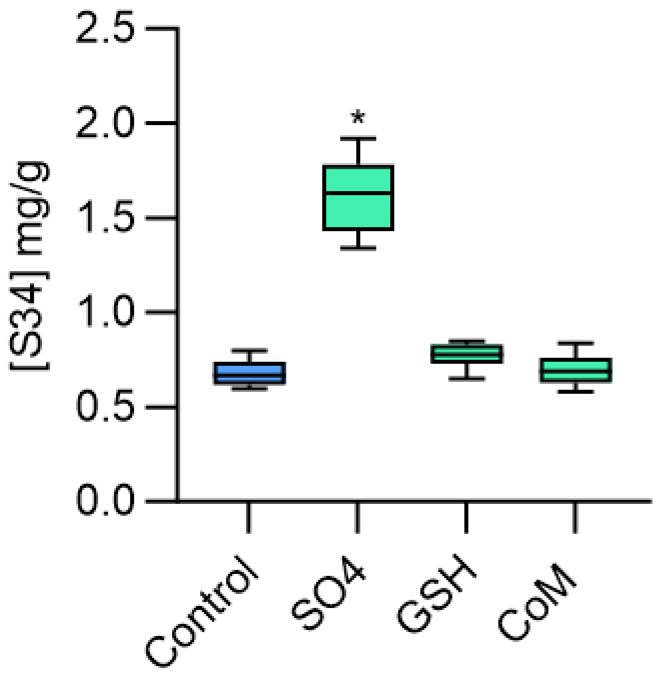

Figure S3. Intracellular Sulfur content of Arabidopsis is unaffected by CoM application. Measurements of internal S_{34} content of Arabidopsis grown on solid media supplemented with 0.5 mM GSH or CoM or 0.3 mM of $MgSO_4 \times 7H_2O$ $n = 3$ biological replicates. All asterisks indicate p-value ≤ 0.05.

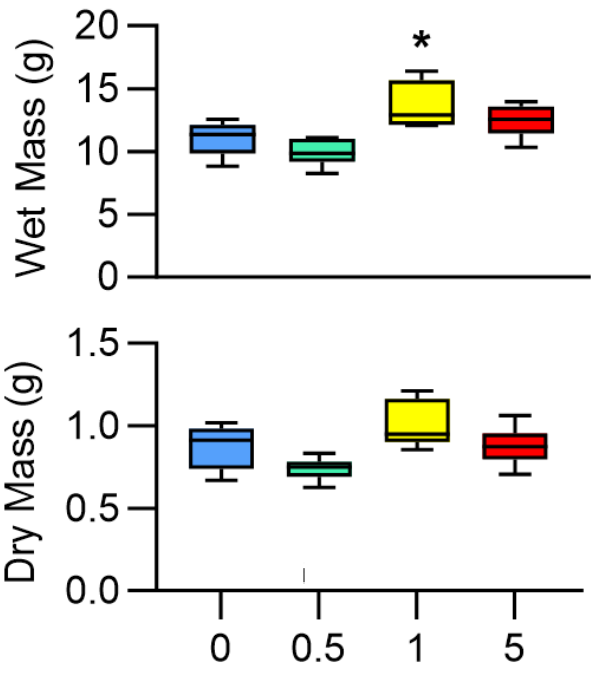

Figure S4. Growth of tobacco is increased by CoM application. 2nd growth trial of tobacco grown to 21 days with spray application of CoM twice weekly with concentrations in mM listed. All asterisks indicate *p*-value ≤ 0.05.

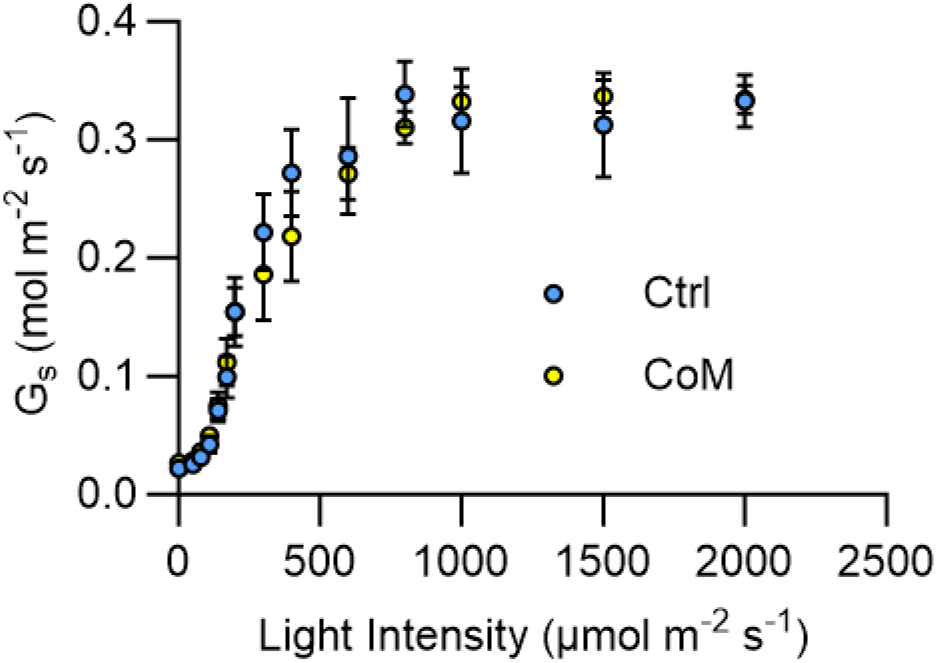

Figure S5. Stomatal conductance of tobacco is unaffected by CoM application. Stomatal conductance (G_s) of tobacco grown to 21 days with spray application of 1mM CoM twice weekly as measured by LiCOR 6800 in response to increasing light. n = 6 biological replicates, lack of asterisks indicate no p-value ≤ 0.05.

Table S1 Statistical analyses of Data

Data	Test	p-value
Fig 1b Ctrl vs. 0.2 mM	Dunnett's multiple comparisons test	<0.0001
Fig 1b Ctrl vs. 0.5 mM	Dunnett's multiple comparisons test	<0.0001
Fig 1b Ctrl vs 1 mM	Dunnett's multiple comparisons test	<0.0001
Fig 1b Ctrl vs 5 mM	Dunnett's multiple comparisons test	<0.0001
Fig 1d Wet mass Ctrl vs GSH	Dunnett's multiple comparisons test	0.0072
Fig 1d Wet mass Ctrl vs CoM	Dunnett's multiple comparisons test	<0.0001
Fig 1d Dry mass Ctrl vs GSH	Dunnett's multiple comparisons test	0.0097
Fig 1d Dry mass Ctrl vs CoM	Dunnett's multiple comparisons test	<0.0001
Wet mass Ctrl vs CoM (1/2 MS)	Dunnett's multiple comparisons test	0.0003
Wet mass Ctrl vs GSH (1/2 MS)	Dunnett's multiple comparisons test	0.1639
Fig 1e -S vs -S+CoM	Dunnett's multiple comparisons test	<0.0001
Fig 2a Ctrl vs 1 mM	Dunnett's multiple comparisons test	0.0090
Fig 2b Ctrl vs 1 mM	Dunnett's multiple comparisons test	0.0060
Fig 2b Ctrl vs 2.5 mM	Dunnett's multiple comparisons test	0.0001
Fig 2b Ctrl vs 5 mM	Dunnett's multiple comparisons test	0.0003
Fig 2c F_v Ctrl vs 1mM	Dunnett's multiple comparisons test	<0.0001
Fig 2c F_v Ctrl vs 2.5 mM	Dunnett's multiple comparisons test	<0.0001
Fig 2c F_v Ctrl vs 5 mM	Dunnett's multiple comparisons test	0.0031
Fig 2c F_m Ctrl vs 1mM	Dunnett's multiple comparisons test	<0.0001
Fig 2c F_m Ctrl vs 2.5 mM	Dunnett's multiple comparisons test	<0.0001
Fig 2c F_p Ctrl vs 1 mM	Dunnett's multiple comparisons test	<0.0001
Fig 2c F_p Ctrl vs 2.5 mM	Dunnett's multiple comparisons test	<0.0001
Fig 2d ϕPSII	2-Way ANOVA (CoM concentration)	<0.0001
Fig 2d ϕPSII	2-Way ANOVA (Time)	<0.0001
Fig 2d ϕPSII	2-Way ANOVA (Interaction of concentration w/ Time)	<0.0001
Fig 2d $PSII_{lightasym}$ Ctrl vs 1 mM	Dunnett's multiple comparisons test	0.0158
Fig 2d $PSII_{lightasym}$ Ctrl vs 2.5 mM	Dunnett's multiple comparisons test	0.0014
Fig 2d $PSII_{lightasym}$ Ctrl vs 5 mM	Dunnett's multiple comparisons test	0.0395
Fig 2d $PSII_{end}$ Ctrl vs 2.5 mM	Dunnett's multiple comparisons test	0.0001
Fig 2d $PSII_{end}$ Ctrl vs 5 mM	Dunnett's multiple comparisons test	0.0032
Fig 2e ϕNPQ	2-Way ANOVA (CoM concentration)	<0.0001
Fig 2e ϕNPQ	2-Way ANOVA (Time)	<0.0001
Fig 2e ϕNPQ	2-Way ANOVA (Interaction of concentration w/ time)	<0.0001
Fig 2e NPQ_{max} Ctrl vs 1 mM	Dunnett's multiple comparisons test	0.0058
Fig 2e NPQ_{max} Ctrl vs 2.5 mM	Dunnett's multiple comparisons test	0.0015
Fig 2e NPQ_{max} Ctrl vs 5 mM	Dunnett's multiple comparisons test	0.0004
Fig 2e NPQ_{rel} Ctrl vs 1 mM	Dunnett's multiple comparisons test	0.0360
Fig 2e NPQ_{rel} Ctrl vs 2.5 mM	Dunnett's multiple comparisons test	<0.0001
Fig 2e NPQ_{rel} Ctrl vs 5 mM	Dunnett's multiple comparisons test	<0.0001
Fig 2e NPQ_{end} Ctrl vs 2.5 mM	Dunnett's multiple comparisons test	0.0030
Fig 2e NPQ_{end} Ctrl vs 5 mM	Dunnett's multiple comparisons test	0.0221
Fig 3a 28 days Ctrl vs 0.5 mM	Dunnett's multiple comparisons test	0.0055
Fig 3a 35 days Ctrl vs 0.5 mM	Dunnett's multiple comparisons test	0.0037
Fig 3b 0 mM vs 2 mM	Dunnett's multiple comparisons test	0.0153
Fig 3c 28 days 0 mM vs 3 mM	Dunnett's multiple comparisons test	0.0060
Fig 3c 35 days 0 mM vs 3 mM	Dunnett's multiple comparisons test	0.0050
Fig 3d Ctrl vs 3 mM	Dunnett's multiple comparisons test	0.0025
Fig 3e Ctrl 1mM	Dunnett's multiple comparisons test	<0.0001
Fig S1 Ctrl vs GSH	Dunnett's multiple comparisons test	0.0856

Fig S1 Ctrl vs CoM	Dunnett's multiple comparisons test	>0.9999
Fig S2 Ctrl vs CoM	Dunnett's multiple comparisons test	<0.0001
Fig S3 Ctrl vs SO_4	Dunnett's multiple comparisons test	<0.0001
Fig S4 Wet Mass Ctrl vs 1mM	Dunnett's multiple comparisons test	0.0130
Fig S5 G_s	2-way ANOVA (Concentration)	0.6817
Fig S5 G_s	2-way ANOVA (Light)	<0.0001
Fig S5 G_s	2-way ANOVA (Interaction of Concentration w/ Light)	0.9686
